# A New Approach to the Formation of Nanosized Gold and Beryllium Films by Ion-Beam Sputtering Deposition

**DOI:** 10.3390/nano12030470

**Published:** 2022-01-29

**Authors:** Sergei A. Sharko, Aleksandra I. Serokurova, Nikolai N. Novitskii, Valerii A. Ketsko, Maria N. Smirnova, Aljawhara H. Almuqrin, M. I. Sayyed, Sergei V. Trukhanov, Alex V. Trukhanov

**Affiliations:** 1 Laboratory of Magnetic Films Physics, Scientific-Practical Materials Research Centre of National Academy of Sciences of Belarus, 220072 Minsk, Belarus; serokurova@physics.by (A.I.S.); novitski@physics.by (N.N.N.); truhanov86@mail.ru (A.V.T.); 2 Kurnakov Institute of General and Inorganic Chemistry of Russian Academy of Sciences, 119991 Moscow, Russia; ketsko@igic.ras.ru (V.A.K.); smirnova_macha1989@mail.ru (M.N.S.); 3 Department of Physics, College of Science, Princess Nourah Bint Abdulrahman University, P.O. Box 84428, Riyadh 11671, Saudi Arabia; ahalmoqren@pnu.edu.sa; 4 Department of Physics, Faculty of Science, Isra University, Amman 11622, Jordan; dr.mabualssayed@gmail.com; 5 Department of Nuclear Medicine Research, Institute for Research and Medical Consultations (IRMC), Imam Abdulrahman Bin Faisal University (IAU), PO Box 1982, Dammam 31441, Saudi Arabia; 6 Laboratory of Single Crystal Growth, South Ural State University, 76, Lenin Av., 454080 Chelyabinsk, Russia

**Keywords:** ion-beam sputtering-deposition, nanosized gold and beryllium films, electric resistance, surface roughness, plasmon resonance

## Abstract

Thin films of beryllium and gold that are several tens of nanometers thick were obtained, for the first time, on silicon and quartz substrates by the ion-beam method with tenfold alternation of deposition and partial sputtering of the nanosized metal layer. Scanning electron and atomic force microscopy indicate the predominant lateral growth of nanosized metal layers along the substrate surface. Optical spectra indicate the suppression of the localized plasmon resonance. The growth of the film occurs under the influence of the high-energy component of the sputtered metal atoms’ flux. The main role in the formation of the nanosized metal film is played by the processes of the elastic collision of incident metal atoms with the atoms of a substrate and a growing metal film. Metal films that are obtained by the tenfold application of the deposition–sputtering of a nanoscale metal layer are characterized by stronger adhesion to the substrate and have better morphological, electrical, and optical characteristics than those that are obtained by means of direct single deposition.

## 1. Introduction

The obtaining of metals in a nanoscale state [1,2,3] and the study of their properties is of decisive importance for modern science and technology and, therefore, is one of the priority directions in the development of materials science.

Continuous films of metals with a thickness from units to tens of nanometers on substrates of various natures, including those that are dielectric, are of considerable scientific and practical interest. In particular, special attention has been paid to gold, associated with its increased resistance to corrosion and high electrical conductivity, which has led to its widespread use in various fields of electronic instrument-making as conductive and protective coatings, as well as for the development of micro-, nano- and magnetoelectronic devices. Nanosized gold films [4,5,6] are capable of transmitting more than half of the incident light flux with an absorption rate of 10–20% [7,8]. This fact allows them to be used as transparent ohmic contacts, transparent protective layers, electrodes in multilayer metal structures [9,10], etc. Another wide application area of these materials is associated with the possibility of their excitation in structures, on their ground elementary oscillations, plasmons, excitons, and magnons. The interaction of these excitations with electromagnetic waves makes it possible to create artificial media with extraordinary properties—for instance, metamaterials—which opens opportunities for the development of devices for plasmonics, nanophotonics [6,7,11,12,13,14,15], and magnetoplasmonics [16,17]. Plasmons can be used for information transmission in computer-integrated circuits that are operating at frequencies of the order of hundreds of terahertz, while ordinary wires have losses already at 10 GHz.

As for beryllium, it has high elasticity and thermal conductivity with a low mass and a relatively high melting point (1278 °C). Beryllium is one of the few transparent materials in the extreme ultraviolet (EUV) and X-ray ranges. Therefore, it is an irreplaceable material in X-ray optics for the windows of vacuum chambers and refractive lenses for synchrotron beams. It can also be used as a component of the highly reflective multilayer mirrors that are used in lithography and solar astronomy [18].

Obtaining continuous and homogeneous transparent gold films [19,20] with a crystal lattice spacing thickness of tens is a difficult problem. The chemical inertness of gold to materials of a different nature determines the predominant interaction being between two gold atoms rather than between the gold and the substrate’s atoms. This gives rise to poor adhesion of the nucleating gold layer to the substrate. The formation of gold–gold bonds on the substrate’s surface prevents a uniform distribution of the nucleation centers and promotes clustering processes in the deposited layer with the subsequent conglomeration of those clusters into gold granules. In the same measure, the aforesaid also concerns the thin beryllium films.

To suppress this granulation in order to obtain denser films, the substrate is usually activated either by heating or by changing the parameters of the ion flux and activation energy. In present vacuum technologies, the substrate is irradiated with a beam of low-energy ions. In this case, more crystallization centers appear and the formation mechanism approaches the corresponding mechanism of the two-dimensional layer-by-layer growth, which is close to the Frank–Van der Merwe growth model [21,22]. However, in the formation of thin films by ion-beam sputtering, deposition conditions that are far from the equilibrium ones that are described by classical thermodynamics are essential. A flux of sputtered atoms with an energy of several tens of electronvolts, which is much higher than the surface activation energy of several eV, hits the substrate. The known growth mechanisms [21,22] for nanometer-thick films are not realized under these conditions.

To ensure high adhesion of the deposited adatoms, a strong interaction between the film and the substrate is also required in addition to the chemically pure substrate. The latter is achieved due to the formation of a chemical bond or the interdiffusion of the materials, which is difficult to implement in the case of nanosized films on an optically transparent substrate; this is especially true of gold, provided its chemical inertion to the substrate. At the same time, the interdiffusion of the materials, which can be initiated, among other things, by high-temperature annealing [23], leads to uncertainty in the determination of the nanometer gold film’s final thickness because of the diffusion of the film–substrate interface region to a depth that is comparable to the thickness of the initial film.

Thus, the problem of obtaining high-quality nanometer-thick gold and beryllium films with high adhesion to the substrate is urgent and is associated with the study of the initial stage of the formation and growth of continuous homogeneous metal films. At the same time, a large difference in their atomic masses (gold is more than 20 times heavier than beryllium) will have a different effect on the film formation process under the argon ion action in the case of the light and heavy atoms, which is the basis for the choice of these two metals.

This article is a continuation of the work on the formation of thin films of metals by the ion-beam method that has been begun elsewhere [7,24,25]. In particular, the development of ideas that are related to the influence of the high-energy component of the sputtered atoms’ flux on the growing metal layer makes it possible to explain adequately the obtaining of high-quality films in the nanoscale state on various substrates.

In this work, the method of ion-beam deposition was used to obtain beryllium and gold films of several tens of nanometers thick on silicon and quartz substrates. An improvement in their physical properties was shown as a result of irradiation of the substrate and the growing film by its own metal atoms with tenfold iteration of the deposition and partial sputtering of the nanoscale metal layer.

## 2. Materials and Methods

### 2.1. Preparation of Materials

**Figure 1:**
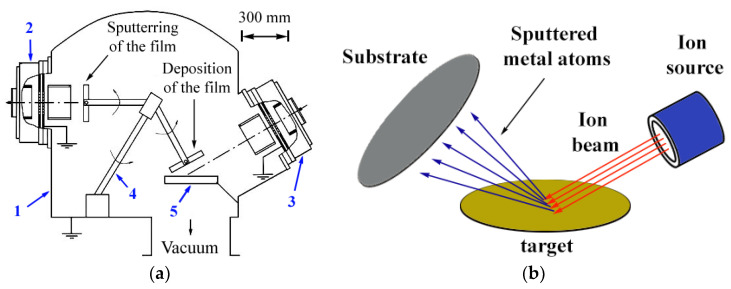
Diagram of the vacuum chamber internal equipment of the ion-beam sputtering–deposition unit (**a**) and the process of ion-beam deposition (**b**). 1. vacuum chamber; 2. and 3. ion sources for sputtering and deposition of the film; 4. substrate rotation mechanism; and 5. target.

The obtaining of metal films was carried out by the method of ion-beam sputtering–deposition (Figure 1a). Before the deposition of the metal layer, the silicon and quartz substrates were cleaned from foreign surface impurities for 120 s by an oxygen ion beam of an energy level of less than 0.3 keV and an ion beam current density of 0.1–0.15 mA/cm^2^. The deposition of a beryllium and gold layer on a previously prepared substrate was carried out by sputtering the corresponding metal target by argon ions with an energy of 1.3 keV and an ion beam current density of 0.1–0.25 mA/cm^2^ at a residual atmosphere pressure that was no higher than 0.2 Pa. The diameter of the ion beam at the exit from the source was 10 cm. To avoid the sputtering of the equipment and the ingress of impurity atoms into the sputtered flow, the ion beam was limited by an aperture to a smaller size in comparison with the target (5–6 cm^2^). To ensure the uniformity of the deposition, the dimensions on the substrates were chosen to equal 4–5 cm^2^. The geometry of the setup, particularly the small distance between the target and the substrate compared to the mean free path of atoms in a working vacuum, made it possible to direct all of the sputtered atoms onto the substrate (Figure 1b). The deposition time for beryllium was 10 min and that for gold was 5 min. With repeated application of the deposition–sputtering operation, the metal layer was deposited under the same conditions for 60 s and the sputtering was carried out by argon ions of the same energies for 30 s. This mode was chosen for the best results based on the analysis of the optical spectra of the films that were obtained at different times of deposition and sputtering. For the purpose of ensuring enhanced adhesion, the first metal layers were sputtered completely before the disappearance of the metal conductivity. The deposition–sputtering cycle was repeated 10 times.

### 2.2. Characterization Methods

The analysis of the surface and cross-sections of the heterostructures was carried out on a scanning electron-ion microscope SEM Helios NanoLab 650 (FEI Company, Hillsboro, OR, USA). Focused gallium ion beams with an energy of 32 keV were used to obtain cross sections. The thicknesses of the metal films were determined from the scanning electron microscopy (SEM) data.

The surface morphology was investigated by contact mode atomic force microscopy (AFM) on a SmartSPM (AIST-NT, Moscow, Russia) scanning probe microscope. For the AFM studies of the individual layers’ surfaces, the Au films with a thickness of 10 nm on silicon were obtained with a single deposition and with a single application of a repeated deposition under identical conditions to those that are described above (deposition for 60 s and then sputtering for 30 s). The root mean square roughness, *R_q_*, of the surface was measured on a characteristic area of the surface of 2 × 2 μm^2^.

The X-ray studies were carried out on a Bruker D8 Advance diffractometer using Cu K_α_ radiation with a wavelength of 1.5405 Å. The X-ray diffraction patterns were obtained in the 2θ angle range of 20–80°. The Inorganic Crystal Structures Database (ICSD) (The University of Southampton, Southampton, United Kingdom) was used in order to identify the X-ray reflections of the corresponding phases.

The electrical studies were carried out using the standard linear four-probe method [26]. The surface resistance *R_s_* of the films that was obtained was measured under thermal cycling from 20 to 130 °C.

The optical reflection and transmission spectra of the gold and beryllium films on the quartz substrates were obtained with the help of a Cary-500 spectrophotometer (StellarNet, Inc. Tampa, FL, USA) in the wavelength range from 400 to 900 nm with an error of no more than 0.3%.

## 3. Results and Discussion

### 3.1. Surface Morphology and SEM

**Figure 2:**
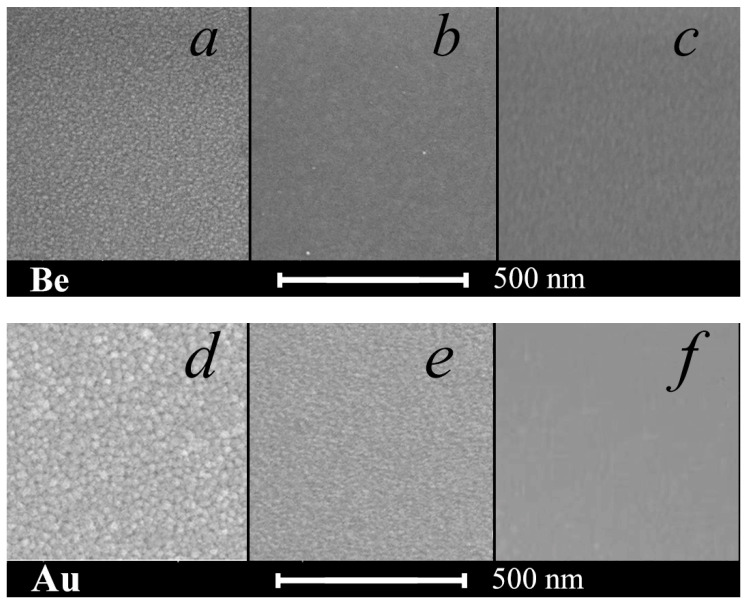
SEM image of the beryllium and gold film surfaces with a single deposition onto the initial silicon surface (**a**,**d**), with a single repeated deposition (**b**,**e**), and with tenfold deposition onto the activated silicon surface (**c**,**f**).

The surface root mean square roughness of a thin metal layer on silicon, as well as the average granular size, decreases upon the transition to the repeated influence of a sputtered atoms beam on the growing film (Figure 2). So, the root mean square roughness *σ* for gold with a single deposition was 1.2 nm, with repeated deposition was 0.8 nm, and with tenfold deposition was 0.3 nm. In the latter case, almost complete granulation suppression of both beryllium and gold films occurred (Figure 2c,f). A beam of sputtered atoms of the high-energy component broke up the metal clusters at the initial stage of their formation. This indicates the suppression of three-dimensional nucleation during the repeated deposition and the transition to a two-dimensional mechanism of a nanoscale metal layer formation with a predominance of the lateral diffusion (along the film plane) over the vertical one.

It is well known [23] that diffusion along the grain boundaries in polycrystalline materials proceeds much more intensively in comparison with a bulk lattice one. The compaction of the granules by an incident flow of fast atoms leads to a decrease in the activation energy of diffusion along the granular boundary, which is parallel to the growing film surface and, thus, to the diffusion rate anisotropy along and across the film’s plane. In this case, the predominant diffusion of adatoms occurs along the boundaries of close-packed granules and, at the same time, deep into the film it occurs with noticeable difficulty.

**Figure 3:**
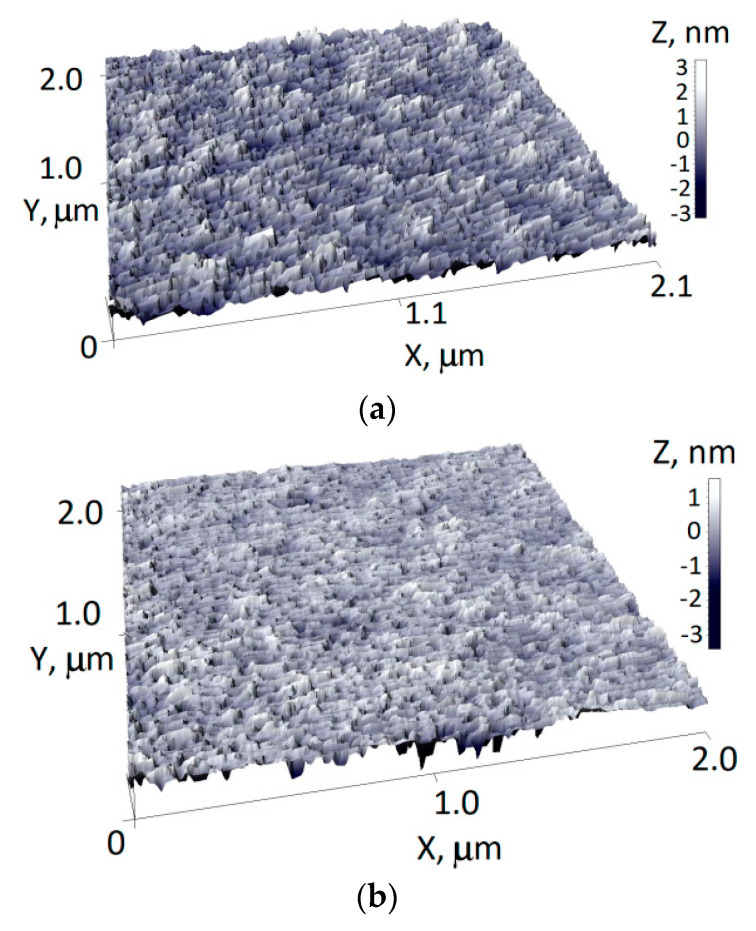
Surface topography of the 10 nm gold layer, obtained by single deposition (**a**) and by the single application of a repeated deposition (**b**).

A decrease in the roughness of the surface relief was noticeable on the topography of the 10 nm gold layers on silicon (Figure 3). So, when passing from a direct single deposition to repeated ones, the maximum height range on a characteristic surface relief of 2 μm in length decreased from 6 to 4 nm with the root mean square roughness decreasing from 0.8 to 0.4 nm.

**Figure 4:**
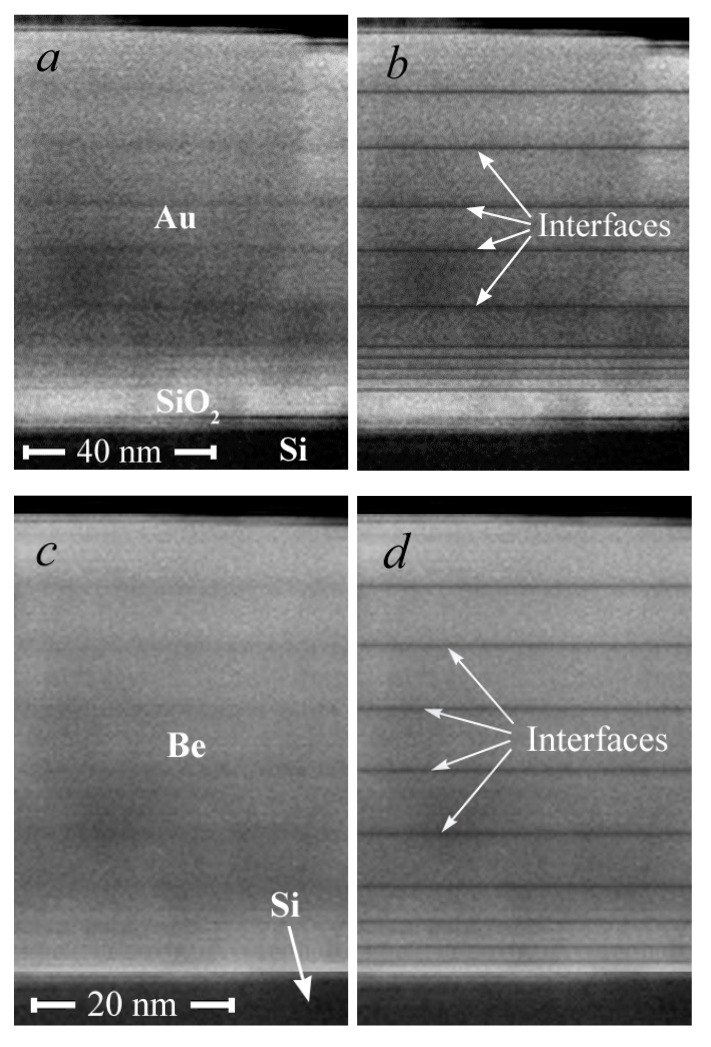
Cross-section SEM image of a 90 nm gold (**a**,**b**) and 45 nm beryllium (**c**,**d**) film on silicon substrate after tenfold deposition. The radiation-modified areas are artificially highlighted during image processing in the right figures (**b**,**d**).

A layered structure is clearly visible in Figure 4, formed as a result of the high-energy metal atoms’ influence first on the substrate and, then, as the metal film grew, on the film itself. The interfaces between the layers are the radiation-modified compacted areas. The goal of our further research is to obtain structures that are more uniform in density, in which dark and light regions will merge into a single unit formation. The thickness of the layers for Au is 9–14 nm and for Be is 4–6 nm. The film grows mainly laterally along the surface, when adsorbed atoms are attached to the layer until it is formed. The growth of the film after each sputtering operation occurs, as at the initial stage, by a mechanism that is close to the two-dimensional growth of Frank–Van der Merwe [21,22], which is possible in the case of the predominance of the lateral diffusion of atoms over the vertical one.

No delaminations were observed in the cross section of the film–substrate interface, and the interface itself was a continuous and even separation surface. That indicates the absence of chemical interaction and foreign phases at the interface. This is evidence of good adhesion of the metal layer to the substrate. The analysis of the cross-section SEM images made it possible to determine the average deposition rate of the gold and beryllium layers to be 0.3 nm/s and 0.15 nm/s, respectively.

Due to the lower mass of Be atoms, as compared to Au ones, they penetrate deeper into the Be matrix than Au atoms do into the Au matrix. As a result, the beryllium film is compacted over the entire depth of penetration of the sputtered atoms. The SEM image is formed in backscattering electrons for which the contrast of the areas is proportional to the average atomic number of the elements that enter into the composition. Therefore, the interface areas in the case of gold (the heavier element) look more contrasted (Figure 4a) than in the case of beryllium (Figure 4c).

### 3.2. X-ray Diffraction

**Figure 5:**
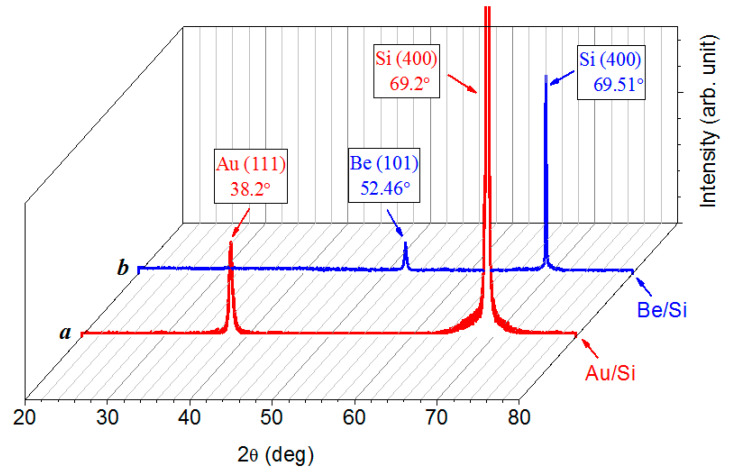
X-ray diffraction pattern of 90 nm-thick gold (a) and beryllium (b) films on silicon substrate obtained by tenfold repetition of deposition (60 s) and sputtering (30 s) processes.

The X-ray diffraction patterns of the gold and beryllium films on a silicon substrate (Figure 5) show that no other phases, except for the metal and the substrate, are contained in the structures that were obtained. The intensity of a single reflection from the substrate, due to its considerable thickness compared to that of the nanoscale film, significantly exceeds the intensity of the strongest reflection from the film. The shift of all of the reflections in regard to the reference data indicates that the metal film and the substrate are in an elastic-stressed state at the interface because of the mismatch of their crystal lattice parameters. The Be–Si and Au–Si structures behave as a unit, where the elastic connection realizes owing to the strong adhesion of the metal film to the substrate.

### 3.3. Electrical Properties

**Figure 6:**
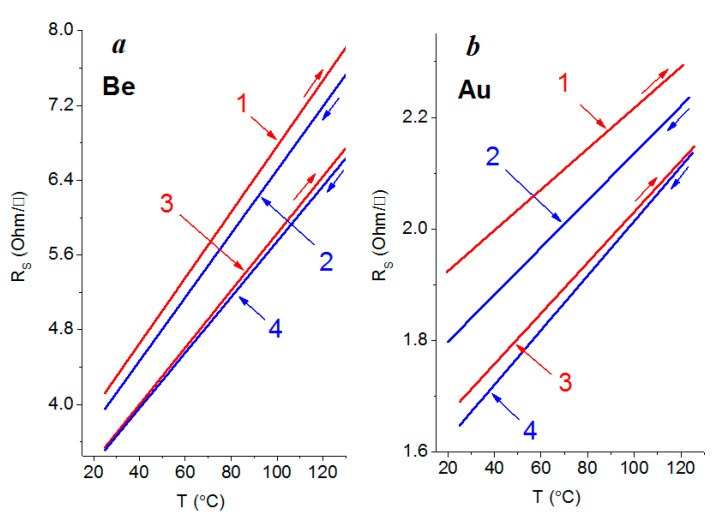
Surface resistance of beryllium (**a**) and gold (**b**) 90 nm thick films with single (1) and (2) and multiple (3) and (4) deposition onto the quartz substrate surface. Increase (1) and (3) and decrease (2) and (4) in temperature. Straight lines were built by a method of least squares.

Electrical measurements show an insignificant decrease in the amount of surface resistance (by 10%) for both the beryllium (Figure 6a) and gold films (Figure 6b) after the tenfold deposition, compared with the same films that were obtained with a single deposition. The scatter of the resistance values of the films that were obtained by multiple deposition also decreases, which indicates an improvement in their thermal stability in the measured temperature range.

For films that are characterized by a physically continuous homogeneous structure, the resistivity *ρ* is due to the volume scattering of conduction electrons by phonons (*ρ*_B_), scattering on the free film surfaces (*ρ*_S_), at the granular boundaries (*ρ*_GB_), on the structural defects (*ρ*_D_), and on the impurities (*ρ*_I_) [27]:

*ρ* = *ρ*_B_ + *ρ*_S_ + *ρ*_GB_ + *ρ*_D_ + *ρ*_I_. (1)

In the region of the existence of continuous homogeneous films, the largest contribution to the electrical resistivity is made by the scattering of electrons on structural defects (mainly on the single vacancies and the clusters that are formed by them inside the granules, as well as on the granular boundaries), since their thickness exceeds the average length *λ*_0_ of the free path of the electrons [27]. For gold the mean free path of the electrons is 37.7 nm [28] and for beryllium it is 48.0 nm and 68.2 nm perpendicularly and parallel, respectively, to the hexagonal axis [28].

In fine-crystalline films, as in our case, the scattering of conduction electrons at the intercrystalline boundaries, rather than on the outer surface of the film as is considered in the Fuchs–Sondheimer theory [29,30], is essential. This case is usually realized in thin single-crystal epitaxial or coarse-crystalline films, in which the crystallite sizes are much larger than the mean free path *λ*_0_ of the electrons in a bulk sample. In our case, as opposed to the abovementioned, the Mayadas–Shatzkes–Janek theory was used as a model of electron transport in fine-crystalline films [31,32]. We applied the results of this theory [33]:

(2) $$\frac{\rho_{MS}}{\rho_{0}}=1-\frac{2}{3}\alpha +3\alpha^{2}-3\alpha^{3}\ln\left(1+\frac{1}{\alpha }\right)$$

where

(3) $$\alpha =\frac{\lambda_{0}}{D}\left(\frac{R}{1-R}\right)$$

Here, *ρ_MS_* is the value of the resistivity due to scattering at the granular boundaries, *ρ*_0_ is the resistivity of the bulk sample, *λ*_0_ is the mean free path in the bulk sample, *D* is the average value of the crystallite size, and *R* is the coefficient of the reflection of the electrons from the internal (intercrystalline) boundaries. The reflection coefficient *R* depends not only on the film’s thickness and the method of obtaining it [33], but it also varies along the perimeter of a single grain from 0.7 to 0.9 [34]. According to [33], the coefficient *R* for gold films that are deposited onto glass substrates by thermal evaporation decreases from 0.8 to 0.6 with an increase in film thickness from 20 to 90 nm. The same values of *R* are accepted also in the given work. As for beryllium films, we accepted values that are much the same as for aluminum [33] at a thickness of 40–50 nm (*R* = 0.5–0.6), as under electric characteristics (specific resistance) aluminum and beryllium are considerably closer to each other than to gold. The average crystallite size *D* was taken from Figure 2a,d for Au 20 nm and for Be 10 nm. After deposition with manifold application of deposition–sputtering, it was impossible to define from Figure 2c,f the crystallite size; therefore, for calculation we accepted it as equal to 1 nm.

As one can see from Table 1, which shows the results of calculating the *α* and *ρ*_GB/_*ρ*_0_ coefficients using Formulas (2) and (3), there is very good agreement with the experimental data (Figure 6), provided that the reflection coefficient is chosen correctly, only for samples obtained by a single deposition. This is naturally due to the presence of a large number of granular boundaries. The specific electrical resistance *ρ*, obtained by the product of the surface resistance *R*_s_ and the thickness of the film, for both gold and beryllium at a temperature of 25 °C is no more than an order of magnitude higher than the corresponding resistivity values from the reference data for bulk materials. Thus, for beryllium after a single and tenfold deposition, it is 37.1 and 31.9 μOhm⋅cm, respectively, and for gold after a single and tenfold deposition, it is 17.3 and 15.2 μOhm⋅cm, respectively. The resistivity *ρ*_0_ for gold and beryllium in the bulk state at room temperature is 2.21 and 3.56 μOhm·cm [28,35], respectively.

However, with a tenfold deposition, a decrease in the granular size (to less than 1 nm) leads to a rapid increase in the number of the granules and the internal boundaries between them. This ultimately leads to a significant, more than an order of magnitude, increase in the parameter *ρ*_GB/_*ρ*_0_. In our opinion, the reason for this is that the structure becomes more homogeneous and the number of internal boundaries decreases with the manifold application of the deposition–sputtering operation of a nanoscale metal layer. In this case, to apply the Mayadas–Shatzkes–Janek model is completely inappropriate. It would be quite fair in this case to use the Fuchs–Sondheimer model, which takes into account the specular reflection of the electrons that are participating in the current transfer from the upper and lower outer boundaries.

### 3.4. Optical Properties

**Figure 7:**
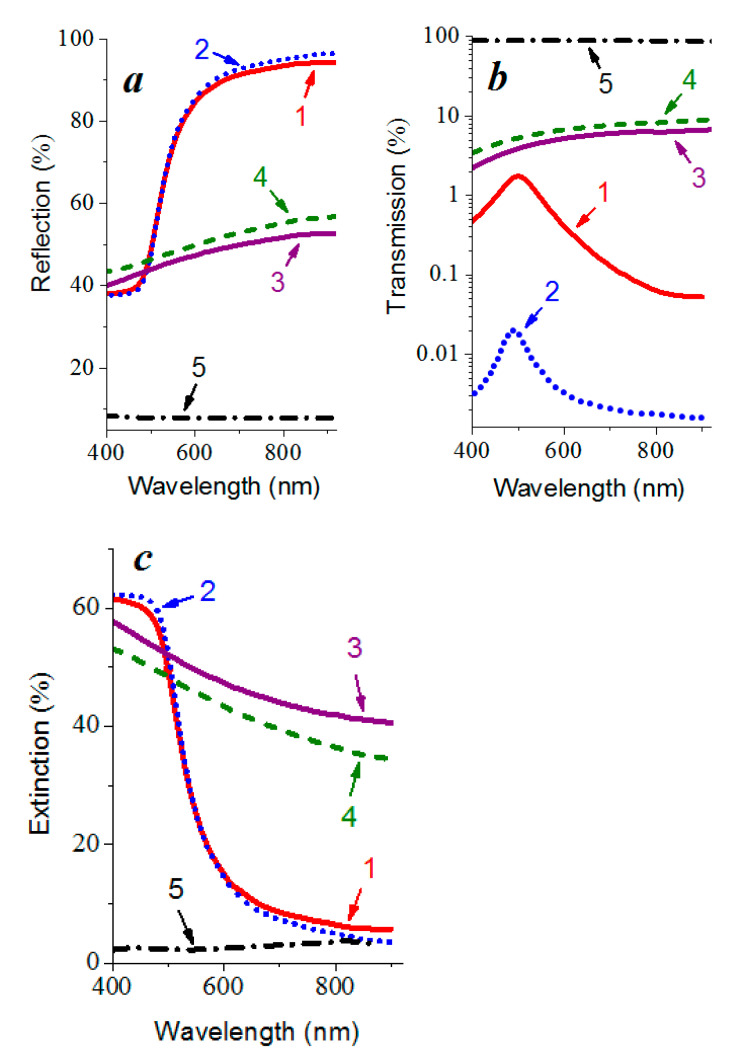
Reflection (**a**), transmission (**b**), and extinction (**c**) spectra of gold (1) and (2) and beryllium (3) and (4) films on quartz obtained by single deposition (1) and (3) and by multiple deposition–sputtering (2) and (4) and the corresponding spectra of quartz (5). Extinction spectra were built on the base of the reflection and transmission spectra.

The optical properties of the beryllium and gold films on quartz substrates in the wavelength range from 400 to 800 nm after applying the operation of tenfold deposition–sputtering did not deteriorate. The analysis of the relationship between the optical coefficients of reflection *R*, transmission *T,* and extinction *A* at a wavelength of 800 nm for the gold and beryllium layers that were obtained both by direct single deposition and by multiple application of the deposition–sputtering operation allows one to conclude that the reflection increases mainly due to a decrease in transmission, rather than the extinction of the waves (Figure 7).

A characteristic feature of the gold and beryllium films that were obtained is a higher reflection coefficient after the tenfold application of the nanoscale metal layer deposition–sputtering, in comparison with the direct single deposition. Thus, at a wavelength of 800 nm for gold films, it increases from 93.5 to 95% (Figure 7a curves 1 and 2), which is quite close to the reflection coefficient for the bulk material [36] that amounts to slightly more than 97% [27]. The reflection coefficient of the nanosized beryllium films has a lower value and increases from 51.7 to 55.3% at the same wavelength.

Light scattering occurs on the surface uniformities that are present in the form of irregular asperities and depressions. On a rough surface, a plane-parallel bundle of rays is scattered in all directions; therefore, a smaller number of rays are collected at the reflection angle and, thus, the diffusivity of the reflection is enhanced. As a result, the detector of the spectrophotometer registers less light intensity. An increase in the reflection coefficient with a decrease in roughness (Figure 7a, curves 1 and 2 or curves 3 and 4) is associated with a decrease in the characteristic size of the surface irregularities. The maximum values of the reflection coefficient that is inherent to bulk materials [36] in the corresponding frequency range are explained by the weakening of the role of the surface in radiation scattering.

The optical reflection *R* and transmission *T* coefficients depend on the microstructure of the films and their values change significantly in the presence of a surface relief. The scattering of radiation with a wavelength *λ* by surface irregularities with a root mean square surface roughness *σ* (at small values of *σ*/*λ*) leads to a relative change in the reflection coefficient by a value [27]:

Δ*R*/*R* ~ (4*πσ*/*λ*)^2^ (4)

Here, Δ*R* is the difference between the reflection coefficients of an idealized surface with zero root mean square roughness and a rough surface of the same material for which *σ* ≠ 0. The values of the *R* coefficient for the films that were obtained by a single deposition and by using tenfold deposition–sputtering at a fixed wavelength of electromagnetic radiation (for example, 800 nm) correlate with the values of the root mean square roughness.

### 3.5. Plasmonic Properties

A sharp increase in the reflection coefficient of the gold films in the wavelength range of optical radiation of 500–550 nm (Figure 7a curves 1 and 2), as well as the absence of the maximum on the extinction curves in the region of 450–520 nm (Figure 7c curves 1 and 2), indicates the suppression of the localized plasmon resonance [37,38,39]. The maximum in the transmission spectra at 500 nm (Figure 7b curves 1 and 2), which is characteristic of both continuous and island films [40], is responsible for the yellowish color of gold.

In the extinction spectrum of thin island-type gold films, near the intrinsic absorption edge that is associated with interband transitions [11], in the wavelength range 450–520 nm, an absorption peak is usually observed corresponding to the localized surface plasmon resonance [41,42]. In the transmission spectra of such films, plasmon resonance appears as a minimum in the region of 500–700 nm [43], which completely disappears just at a thickness of 10 nm. This minimum shifts to longer wavelengths (a red shift) with increasing film thickness becomes less pronounced and disappears at a thickness corresponding to a continuous film. In [44], the minimum at 520 nm in the transmission spectra of the gold particles in thin-film gold–polytetrafluoroethylene composites shift to longer wavelengths with an increase in the annealing temperature and, hence, with an increase in the nanoparticles’ sizes. In our case, the minimum transmission in this wavelength region was not observed (Figure 7b curves 1 and 2).

The absence of the characteristic features that are associated with the localized plasmons’ excitation in the optical spectra of gold films corresponds to the homogeneous structure throughout the thickness, in which there are no internal intergranular boundaries, and, therefore, the film responds to electromagnetic radiation as a single formation. As for beryllium, it does not exhibit resonance properties in the considered spectral range.

### 3.6. Formation Mechanism of Nanosized Metal Films

The features of the sputtering processes and, consequently, the obtaining of thin films are determined by the kinetics of collisional processes on the grounds of energy and momentum conservation laws [45]. The maximum fraction of the transferred kinetic energy from an incident atom to a resting one in one collision in a case of a central absolutely elastic impact is determined by the ratio of the masses of the incident *M*_1_ and resting *M*_2_ atoms:

(5) $$\gamma =\frac{4M_{1}M_{2}}{{\left(M_{1}+M_{2}\right)}^{2}}$$

The energy distribution of the sputtered metal atoms has a continuous spectrum up to the maximum energy *E*_max_ [46]. According to the theory of linear cascades that was proposed by Falcone and Sigmund and taking into account the pair collision and role of the surface in sputtering [47], the maximum energy of the sputtered particles linearly increases with the increase in the primary particles’ energy *E*:

*E*_max_ = *AE* − *U*, (6)

where *A* = γ(1 − γ) is the maximum fraction of the energy of the primary particles that can go to recoil atoms that were sputtered after one collision; *U* is the sublimation energy. As for the energy of the incident argon ions corresponding to the experiment—1.3 keV, the maximum energy of the sputtered atoms was about 300–320 eV (Table 2).

The number of sputtered particles d *d*^2^*S*, emitted in a unit solid angle *d*Ω and possessing energies in the range from *E* to *E* + *dE*, is well approximated by the distribution function [47]

(7) $$\frac{d^{2}S}{d\Omega \;dE}=\frac{M_{2}}{2}\frac{E}{{\left(E+U\right)}^{3}}\left(1+\frac{E}{E+U}\right).$$

**Figure 8:**
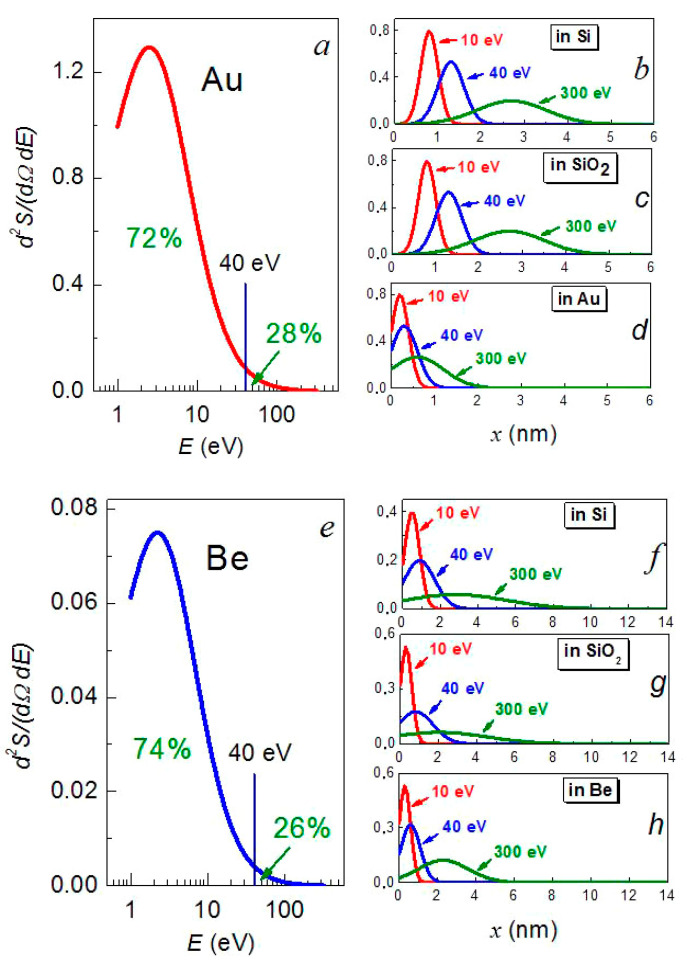
Energy distribution of the sputtered gold (**a**) and beryllium (**e**) atoms according to (7) on a logarithmic scale along the energy axis. Depth distribution of the sputtered gold atoms with energies of 10, 40, and 300 eV in silicon (**b**), quartz (**c**), and gold (**d**) and beryllium atoms with energies of 10, 40, and 300 eV in silicon (**f**), quartz (**g**), and beryllium (**h**).

This function has an asymmetrical shape with the slowly decreasing right branch at large energies (Figure 8a,e).

To explain the formation of a high-quality continuous metal film on the substrate’s surface, the flux of the sputtered metal atoms can be conditionally divided into two parts: main and high-energy [24,25]. The main part consists of the atoms with an average energy that is approximately equal to the sublimation energy *U* (for beryllium and gold ≈ 3–4 eV/atom) and the high-energy part includes the atoms with an energy of more than 40 eV, i.e., an order of magnitude higher than *U* [46,48]. The integration of the energy spectrum of the sputtered atoms (7) over energy up to the highest energy *E*_max_ gives an estimate of the relative number of the sputtered atoms with energies up to 40 eV of a little more than 70% (Figure 8a,e). The rest is made up of atoms with energies from 40 eV to the maximum possible *E*_max_, with the most energetic of them being less than 2.5%.

The depth of a metal atom’s penetration into the substrate is roughly determined by its initial energy and the ratio of its mass to the mass of the substrate’s atom. According to estimates that were carried out by the Monte Carlo method using the SRIM-2013.00 software package (www.srim.org (accessed on 25 January 2022)) [49], the majority of the sputtered beryllium atoms with energies up to 40 eV penetrated into the silicon substrate to a depth of no more than 0.8 nm (Table 3, Figure 8f), which is no more than one parameter of its crystal lattice (*a*[Si] = 5.4307 Å [50]). The gold atoms with the same energy penetrated into the silicon substrate to a depth of 1.3 nm (Table 3, Figure 8b), i.e., no more than two parameters of the crystal lattice. They formed a metal layer on the substrate’s surface.

The fastest beryllium and gold atoms were able to penetrate into the substrate to a depth of 2.9 nm (up to five lattice spacings) (see Table 3). However, most of them were distributed in the substrate at a depth of no more than one or two lattice spacings in the form of point defects.

The penetration depth of the gold atoms of high energies to the maximum possible energy in the gold matrix was 0.6 nm (Table 3, Figure 8d), i.e., no more than one lattice spacing of gold (*a*[Au] = 4.0781 Å [50]). In beryllium, the depth of penetration of the beryllium atoms was much greater, up to 2.5 nm (Table 3, Figure 8h), which amounts to 7–10 lattice spacings of beryllium (beryllium crystallizes in the hcp lattice with the parameters *a*[Be] = 2.286 Å and *c*[Be] = 3.584 Å [50]). This allows one to assume that a mechanism of the densification of metal films by their own atoms is possible only in the case of the deposition of a gold film rather than that of a beryllium film. This statement is confirmed by the analysis of the SEM image of the cross-section, in which, in the case of gold, the interface regions modified by irradiation with high-energy atoms are more visible (Figure 4a,c).

Redeposition of a metal layer under the above conditions allows at least a twofold increase in the number of embedded metal atoms at the expense of a high-energy component of the sputtered atoms’ flux. For this purpose only, the first metal layers are almost completely removed from a substrate’s surface and all of the subsequent layers are removed only partially. Removal of the surface metal layer also prevents the granulation of films of less than 2 nm. High energy atoms pass through the remaining part of the layer after sputtering at the substrate–film interface and reach the near-surface layer of the substrate, forming point defects there in addition to the existing point defects that were introduced during the previous deposition. They lead to improved adhesion [24,25,48] due to the formation of additional physical bonds between the implanted and deposited metal atoms. This ensures the formation on the substrate of a continuous metal layer with a thickness of several atomic layers and the subsequent nanosized film deposition of the same metal on its surface.

With the same masses of the atoms, realized when the growing film is irradiated with its own atoms, the maximum energy transfer coefficient γ (5) is equal to unity. This means that in the case of a central impact, the incident atoms interchange energy with the atoms that are in the near-surface layer and the knocked-out atoms travel a distance of no more than the penetration depth (see Table 3), i.e., they remain in the metal layer. The incident atoms compact the metal layer and bring in additional energy, which is redistributed in this layer and spent on the suppression of granular formation. All of the above leads to an improvement in the surface’s morphology, which can be seen when comparing the results of the AFM studies of nanosized metal films of the same thickness, obtained under different conditions of ion-beam deposition.

Special attention should be paid to the issue concerning the process of the sputtering of metal layers by argon ions and by high-energy metal atoms. When a metal layer is bombarded with ions, two competing processes occur: its sputtering and the introduction of ions into the metal matrix. According to the model calculations that were performed using the SRIM-2013.00 software product, the sputtering yield by argon ions with an energy of 1.3 keV is 0.6 for beryllium and 4.5 for gold. The sputtering yield *Y* is related to the sputtering rate of the layer

(8) $$v=\frac{j}{eN_{A}}\frac{\mu }{\rho }Y,$$

where *j* is the ion current density in the section perpendicular to the surface of the sputtered film; *μ* is the molar mass of the atoms of the sputtered material; *ρ* is the density of the sputtered material (1.848 and 19.3 g/cm^3^ for beryllium and gold, respectively [50]); *e* = 1.6 × 10^−19^ C is the elementary electric charge; *N_A_* = 6.022 × 10^23^ mol^−1^ is Avogadro’s constant. Under the conditions of the experiment that was carried out in the present work at an ion beam current density of 0.25 mA/cm^2^, the rates of sputtering by argon ions for beryllium and gold are ≈0.1 and 1.2 nm/s, respectively and the penetration depth of the ions in them reaches 4.3 and 2 nm.

A comparison of the sputtering rates of the metal layer and the penetration depth of the argon ions shows that, at an ion energy of 1.3 keV, sputtering processes prevail over ion penetration processes. Indeed, the rates of sputtering and the deposition of the layers are comparable with each other and the depth that was reached by Ar^+^ ions does not exceed the thickness of the metal layer that was sputtered in one cycle. As regards the impact of own-metal atoms on the growing film, the sputtering process with their participation is not of great importance since, firstly, the sputtering yield for them is very small even at energies of 300 eV and, secondly, the number of high-energy atoms is relatively small (Figure 8a,e).

## 4. Conclusions

This work demonstrates a new approach to the formation of continuous homogeneous nanosized metal films with predominantly lateral growth due to the stimulation of the nucleating layer by its own flux of high-energy adatoms.

The use of the technique of the manifold repetition of deposition—sputtering operations makes it possible for the high-energy part of the deposited metal atoms’ flux to repeatedly affect the film that is being formed. This creates conditions for the predominance of the lateral diffusion of adatoms along the granular boundaries over the diffusion along the direction of the film’s growth. This ensures the layer-by-layer growth of nanosized films of beryllium and gold due to the formation of strong adhesion of the metal layer to the substrate. These films are continuous and are characterized by a high degree of homogeneity due to the complete suppression of granulation, as evidenced by studies using scanning electron microscopy methods. On the comparison of a direct single deposition to multiple ones, the root mean square roughness of the surface of gold and beryllium films in a typical relief section of 2 × 2 μm^2^ decreases to a level of less than 1 nm and the maximum height range also decreases.

The shift of the reflections of both the beryllium and gold and the silicon substrate in the X-ray diffraction patterns of the Be/Si and Au/Si structures proves the presence of a strong elastic bond between the metal film and the substrate, which appears due to the strong adhesion of the metal film to the substrate.

The decrease in surface resistance (by no more than 10%) with a simultaneous decrease in the values scattering on the temperature dependence for the gold and beryllium films that were obtained by manifold deposition testifies in favor of this process improving the films’ thermal stability.

There is a satisfactory agreement between the theory of electron transport in fine-crystalline films that was suggested by Mayadas—Shatzkes—Janek with the present experimental data, provided the correct choice of the coefficient of reflection of conduction electrons from the internal granular boundaries, only for the nanoscale films that were obtained by single deposition. In the case of a decrease in the size of granules (to less than 1 nm) or to their complete disappearance leading to an increase in the number of granular boundaries (with manifold deposition), this theory is not applicable.

The reflection coefficient of the nanoscale gold films at a wavelength of optical radiation of 800 nm increased from 93.5 to 95% upon passing to manifold deposition, which is quite close to the reflection coefficient for a bulk material, which is slightly more than 97%. The reflection coefficient of the nanosized beryllium films had lower values at the same wavelength and increased from 51.7 to 55.3%. An increase in the reflection coefficient in the spectral range of 500–550 nm and the absence of an extinction peak of the nanometer gold films in the spectral range of 450–520 nm indicates the suppression of localized plasmon resonance. This is due to a decrease in the number of the internal intergranular boundaries and an increase in the uniformity of these films.

## Figures and Tables

**Table 1:** The results of calculating the *α* and *ρ*_GB/_*ρ*_0_ coefficients using Formulas (2) and (3).

Metal	Conditions of Deposition	*λ*_0_, (nm)	*D*, (nm)	*R*	*α*	*ρ*_GB_/*ρ*_0_
Au	single	37.7	20	0.8	7.5	6.4
				0.7	4.4	3.8
				0.6	2.8	2.6
Au	tenfold	37.7	1	0.8	150.8	125.7
				0.7	87.9	73.3
				0.6	56.6	47.1
Be	single	48.0	10	0.6	7.2	6.1
				0.5	4.8	4.1
Be	tenfold	48.0	1	0.6	72	60
				0.5	48	40

**Table 2:** Molar mass *M*, sublimation energy *U*, impact parameters γ and *A*, threshold *E_th_ = U/A*, and maximum energy *E*_max_ at a central elastic collision of an argon ion with an energy of 1.3 keV with an immobile metal atom.

Metal	*M* (g/mol)	*U* (eV)	*γ*	*A*	*E*_th_ (eV)	*E*_max_ (eV)
**Be**	9.012	3.48	0.6	0.24	14.5	308
**Au**	196.967	3.92	0.56	0.246	15.91	316

**Table 3:** Results of modeling the penetration depth of beryllium atoms in the silicon, quartz, and beryllium matrices and gold atoms in the silicon, quartz, and gold matrices with energies from zero to *E*_max_ = 320 eV, using the SRIM program.

Energy of Sputtered Atoms (eV)	Penetration Depth (nm)					
	of Beryllium Atoms			of Gold Atoms		
	In Silicon	In Quartz	In Beryllium	In Silicon	In Quartz	In Gold
5	0.3	0.2	0.2	0.6	0.6	0.2
10	0.4	0.3	0.3	0.8	0.7	0.2
40	0.8	0.7	0.6	1.3	1.2	0.3
100	1.4	1.2	1.0	1.8	1.7	0.4
300	2.8	2.0	2.3	2.7	2.6	0.6
320	2.9	2.6	2.5	2.8	2.7	0.6
